# Synchrotron X-ray imaging of soft biological tissues – principles, applications and future prospects

**DOI:** 10.1242/jcs.261953

**Published:** 2024-10-23

**Authors:** Jonas Albers, Angelika Svetlove, Elizabeth Duke

**Affiliations:** European Molecular Biology Laboratory, Hamburg Unit c/o DESY, Notkestraße 85, 22607 Hamburg, Germany

**Keywords:** Phase-contrast imaging, Soft tissue imaging, Synchrotron, Virtual histology, X-ray imaging

## Abstract

Synchrotron-based tomographic phase-contrast X-ray imaging (SRµCT or SRnCT) is a versatile isotropic three-dimensional imaging technique that can be used to study biological samples spanning from single cells to human-sized specimens. SRµCT and SRnCT take advantage of the highly brilliant and coherent X-rays produced by a synchrotron light source. This enables fast data acquisition and enhanced image contrast for soft biological samples owing to the exploitation of phase contrast. In this Review, we provide an overview of the basics behind the technique, discuss its applications for biologists and provide an outlook on the future of this emerging technique for biology. We introduce the latest advances in the field, such as whole human organs imaged with micron resolution, using X-rays as a tool for virtual histology and resolving neuronal connections in the brain.

## Teaser

**Summary:** In this Review, we describe the basics of synchrotron-based tomographic phase-contrast X-ray imaging, discuss its applications for biologists and provide an outlook on the future of this emerging technique for biology.

## Introduction

X-ray absorption by materials is directly related to the atomic number (see Glossary) of the elements that make up the material. As a consequence, until recently, X-ray imaging was rarely used to study biological soft tissue because these samples are made up of ‘light’ elements and thus do not absorb enough X-rays to generate an image with sufficient contrast to resolve structures. Heavier biological materials, such as bone and teeth, absorb more X-rays making them more compatible with absorption-based X-ray imaging – as is the case in the traditional hospital setting. X-radiation forms one band on the electromagnetic spectrum and comprises waves with an energy between 0.1 keV (100 eV) and ∼200–300 keV, although there is no universal definition of an X-ray. At the lower end of the energy band the X-rays are termed ‘soft’, and they have minimal penetration power when compared to the high energy (‘hard’) X-rays and are stopped by air. X-rays that are used for the imaging of biological tissues typically fall within the energy range of 10–40 keV depending on the type of imaging. The majority of implementations employ X-rays of a single energy (monochromatic), although a wider energy range (white or pink beam) can also be used. Hard X-rays can be easily generated using laboratory-based sources and indeed, until recently, the majority of biological X-ray imaging was done using these sources where experiments took many hours (or days) and the generation of sufficient contrast within a tissue sample was a significant challenge.

**Glossary**

**Absorption:** the absorption of electromagnetic radiation (the photon energy) by the electrons bound within the atoms.

**Atomic number:** the number of protons in the nucleus of the atom. This gives the weight of the element. The atomic number plays a key role in how the atom interacts with the X-rays.

**Attenuation:** the reduction of the intensity of an X-ray beam as it traverses matter. The reduction might be caused by absorption or by deflection (scatter) of photons from the beam and can be affected by different factors, such as beam energy and atomic number of the absorber.

**Beamline:** refers to the trajectory of the beam including the overall construction of the path segment (guide tubes, diagnostic devices) along a specific path of an accelerator facility. When talking about SRµCT the term “beamline” is also used to describe the experimental end-station – the imaging setup itself.

**Brilliance:** the amount of concentrated photons (flux) that traverses through a defined volume (e.g. sample) in a given time. Synchrotron scientists use brilliance to describe the flux per unit volume.

**Coherence:** refers to the oscillating X-rays being in phase – i.e. when a beam of light has all the photons at the same point on the oscillation curve at any one time. If a wave is coherent, it can produce detectable wave-like effects such as interference.

**Collimation:** a collimated beam of light or other electromagnetic radiation has parallel rays, and therefore will spread minimally as it propagates. At a synchrotron the radiation produced is highly collimated due to its creation by the acceleration of a relativistic (travelling at the speed of light) electron.

**Contrast transfer function algorithm (CTF):** phase retrieval algorithm to extract phase information in the holographic phase contrast regime. Requires projection data at multiple propagation distances (typically four).

**Deposition of dose:** the amount of radiation incident on the sample that is actually absorbed by the sample.

**Dose:** the amount of radiation incident on the sample.

**Edge detection (direct-contrast regime):** the edge detection or direct-contrast regime describes a phase contrast imaging setup with a rather small propagation distances, where diffraction is reduced to edge enhancement and the original image can still be recognised quite well.

**Electron volt (eV):** the measure of the energy photon of a photon – an electron has an energy of 1eV if it has been accelerated by a potential difference of 1V. It is a measurement unit popular amongst synchrotron scientists.

**Holographic phase-contrast regime:** describes phase-contrast imaging setups with larger propagation distance where multiple oscillatory variations, so-called fringes, can be observed.

**Phase retrieval:** describes an algorithm for extracting quantitative phase information from intensity measurements such as X-ray projections.

**Projection:** a 2D radiographic image, a single image in a tomographic acquisition.

**Propagation distance or sample-to-detector distance (SSD):** the propagation distance describes the physical distance between the samples and the detector. For phase contrast imaging it is a crucial parameter since after passing through the sample the intensity of the phase-contrast fringes increases with the propagation distance due to self-interference of the waves, leading to a stronger phase signal.

**Reconstruction:** tomographic reconstruction refers to the process of solving the inverse problem of generating a 3D volume out of a finite number of projection images.

**Refractive index:** the refractive index determines how much the path of light is bent, or refracted, when entering a material. In contrast to visible lights X-rays refract much less when traveling through matter, resulting in refractive indices lower than but very close to 1.

**Synchrotron light source:** a synchrotron light source is a source of electromagnetic radiation (e.g. X-rays) produced by a storage ring. Particles, usually electrons, are accelerated and “bend” into a circular path by strong magnetic field, thereby emitting electromagnetic radiation.

**Tomography:** in the case of X-rays, tomography always refers to the acquisition of projection images at different angles (≥180° radial coverage) with subsequent tomographic reconstruction into a 3D volume.

**VOI:** volume of interest.

**Voxel:** a 3D volumetric pixel (one data point in a reconstructed volume).

**White, pink, polychromatic or monochromatic beam:** an X-ray beam comprising photons of just one energy is termed a monochromatic beam. If the energy spread of the X-ray beam is broadened slightly, the beam is termed pink. An X-ray beam with a wide energy range can be termed both white and polychromatic.

**Fig. 1.**
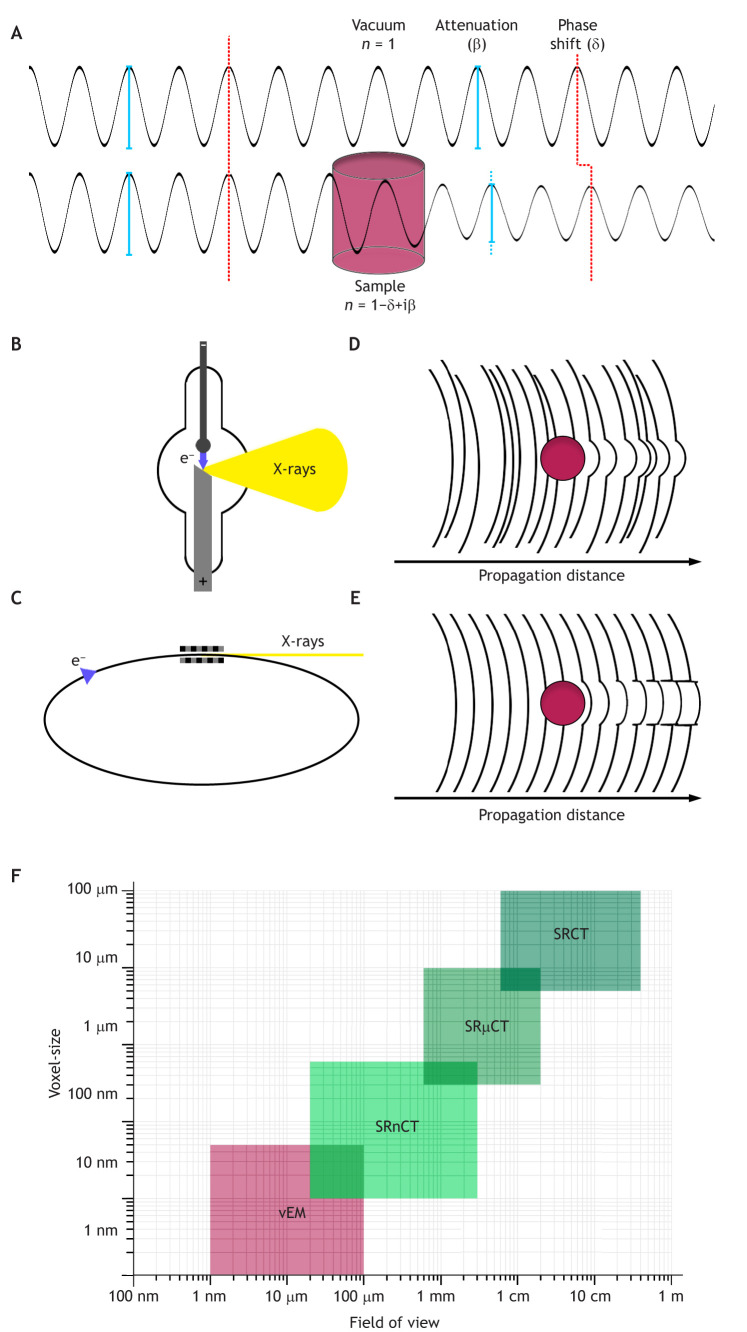
**Principles of propagation-based phase-contrast imaging and technique comparison.** (A) An electromagnetic wave (X-rays) propagating through a sample with its complex refractive index *n*=1−δ+iβ, where *n* is the refractive index, δ accounts for the phase contribution and β for the absorption. (B,C) A standard X-ray tube produces X-rays in a cone beam geometry (B), whereas a synchrotron light source produces a quasi-parallel beam (C). (D) Wavefront scheme of an incoherent X-ray beam created by an X-ray tube. (E) Wavefront scheme of a spatial coherent synchrotron X-ray beam. After passing through the sample the intensity of the phase-contrast fringes increases with the propagation distance due to self-interference of the waves. (F) SRµCT and SRnCT methods can analyse samples ranging from a few microns in size up to human-sized samples using voxel sizes down to a few nanometres. Currently only volume electron microscopy (vEM) can create 3D datasets with higher resolution. vEM techniques include serial section electron tomography (ssET), serial section TEM (ssTEM), serial blockface SEM (SBF-SEM), array tomography and focused ion beam SEM (FIB-SEM). For more details please refer to Peddie et al. (2022).

However, in recent years, advancements in synchrotron hard X-ray imaging have sought to overcome this issue. In this Review, we outline the theory behind synchrotron hard X-ray imaging, focusing on propagation-based phase-contrast tomography (see Glossary). We discuss the principles underlying this technique and how it can be utilised by biologists to further their imaging-based research. We highlight the recent advancements in the field including the imaging of whole organs at a micron resolution, mapping of the neuronal connectome and correlative imaging approaches. It is worth noting that this technique shares many names and abbreviations (Table 1). The original concept of phase-contrast imaging using visible light was created by Frits Zernike over 80 years ago (Zernike, 1942, 1955). X-ray phase-contrast imaging using a crystal interferometer was proposed by Ulrich Bonse and Michael Hart (Bonse and Hart, 1965) in the 1960s and later applied in biology by Atsushi Momose (Momose et al., 1996). The principles of free propagation phase-contrast imaging were conceptualised by Anatoly Snigirev et al. in 1995 (Snigirev et al., 1995). Since then, a multitude of techniques has been developed building on this concept and a huge number of experiments performed at different synchrotron facilities. Although the methods do differ in the X-ray beam geometry, propagation distance, number of distances, energy, coherence (see Glossary) and detector configurations, the principal technology remains the same (Fig. 1). For the purpose of this Review, we use the abbreviation SRµCT or SRnCT to refer to synchrotron radiation micro- or nano-computed tomography, respectively.

**Table 1.**
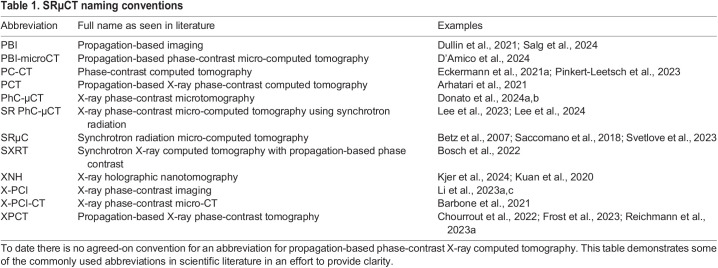
SRµCT naming conventions

### Principles of synchrotron hard X-ray imaging

A synchrotron is a type of particle accelerator (storage ring) that generates X-rays via the acceleration of charged particles (typically electrons); the constant acceleration required to maintain the particles within the confines of the approximately circular ring results in the generation of X-radiation. These synchrotron X-rays can be precisely tuned in energy by the insertion of magnets into the storage ring, and the resulting beams of X-rays are directed down straight-line pipes (beamlines; see Glossary) while the charged electrons continue their trajectories around the ring (Clarke, 2004). There are three features of a synchrotron X-ray beam that are key to its enhanced imaging capabilities: it has a high degree of coherence, is highly collimated and has high brilliance. Coherence refers to the oscillating X-rays being in phase (Lengeler, 2001), i.e. when a beam of light has all the photons at the same point on the oscillation curve at any one time. Typically, X-ray beams from a lab-based source are not coherent, although developments are taking place to increase the degree of coherence of the beams (Dierks et al., 2023; Otendal et al., 2008; Töpperwien et al., 2018a; Tuohimaa et al., 2007). Collimation refers to the degree of beam divergence. High collimation means that light rays are parallel to one another (see Glossary) and thus, the beam does not significantly increase in size as it travels. Brilliance describes the intensity and directionality of an X-ray beam. Coherence and high collimation enable imaging of biological samples without the need to stain the samples to improve the contrast, while high brilliance results in the rapid acquisition of data.

Fundamental to every imaging modality is that contrast is required for image formation. With X-rays, the contrast can be generated either by differential absorption of the X-rays by the sample or by a shift in phase, resulting from variations in the refractive index (see Glossary) within the sample. The former method is typically termed ‘attenuation’ (see Glossary) or ‘absorption’ imaging and the latter ‘phase-contrast’ imaging. In soft tissue samples, the refractive index varies across tissue interfaces (e.g. muscle versus connective tissue), cell boundaries and even intracellular features (e.g. nucleolus within the nucleus) (Momose et al., 1996). Additionally, some structures provide an enhanced contrast due to a natural air–tissue interface, as in the case of empty vessels and bronchi (Kitchen et al., 2017). These features generate tiny phase shifts in the incident X-ray beam (Fig. 1A); when an X-ray beam has a significant level of coherence, the amount of change in the phase can be measured provided the wave front is allowed to self-interfere. One of the keys to successfully exploiting these tiny signals for imaging purposes has been the development of suitable detectors with small enough elements to be able to record these minute changes in phase. This was not possible with the older, lower resolution detectors. Small element detectors typically utilise a scintillator to convert the X-rays into visible light, which is what is actually recorded by the detector. There are direct conversion detectors, which resolve the X-ray signal directly; however, to date they exist mostly with large elements resulting in 20–100 µm voxels (see Glossary). Typically, the imaging application will dictate the detector choice.

Besides the advantage of coherence, synchrotron sources offer extremely high brilliance – they generate a lot of light (photons) into a very narrow pencil beam. A large number of photons enables much faster imaging by decreasing the exposure time from hours, in a typical laboratory source, to minutes on a synchrotron source (Albers et al., 2024). The short data acquisition times mean that the sample is less likely to move during data collection, which is another factor that contributes to the quality of the final data being higher than what can be obtained with a laboratory source. More importantly, this has huge advantages in throughput; quick data collection permits studying large cohorts of samples required to draw tight, statistically justified conclusions.

### The basics of phase-contrast X-ray tomography

X-rays penetrate matter, enabling details within the materials to be imaged non-destructively – i.e. you can see inside something without cutting it open. This is in contrast to, for example, volume electron microscopy (vEM), which requires the sample to be sliced very thinly during or before the imaging process. To be able to sufficiently penetrate samples of a relevant biological size, hard X-rays are used. Hard X-rays have a high photon energy and therefore a short wavelength. Measurement of the phase differences across interfaces in biological tissue directly at the sample position is very difficult, owing to a minimal modulation of the phase signal by the sample. In propagation-based phase-contrast imaging, the distance between the sample and the detector (the propagation distance) is intentionally increased to let the X-ray wave front self-interfere and produce modulations (fringes), which can be measured at the detector position (see Box 1 for the underlying physical concepts) (Fig. 1D,E). One of the challenges is to establish the optimal sample-to-detector distance(s) for data collection. Factors to take into consideration include the shape of the X-ray beam [i.e. cone (focused) beam or parallel beam, see Fig. 1B,C], the energy of the X-rays, the sample size and the desired resolution. Once the projection (see Glossary) images are recorded, information on both the phase and the absorption are contained inside. The phase information is extracted using a process called ‘phase retrieval’ (see Glossary). Currently, there are two main branches of propagation-based phase-contrast imaging: edge detection and holographic phase contrast (see Glossary). Edge detection mode (also called ‘direct-contrast regime’) acquires a tomographic dataset at a single propagation distance, which results in projections with strongly enhanced edge contrast. The phase is retrieved using algorithms based on transport of intensity equations (TIE), commonly referred to as ‘Paganin phase-retrieval’ (Nugent et al., 1996; Paganin et al., 2002). In holographic phase contrast, a series of images is recorded at different propagation distances, typically four, where each distance is most sensitive to a specific range of spatial frequencies. The holographic mode requires longer propagation distances than edge detection. This phase retrieval is usually performed according to the contrast transfer function method (see Glossary) (Cloetens et al., 1999a,b; Zabler et al., 2005). Other methods of phase retrieval exist and are fundamentally based on the methods described above (for further reading refer to Salditt et al., 2017).

**Box 1. Physics concepts underlying X-ray imaging**

X-ray imaging via absorption contrast relies on image generation via the Beer–Lambert law, which in its simplest form is:
(1)

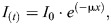

where *I*_0_ is the incident X-ray intensity and *I*_(*t*)_ is the transmitted X-ray intensity through a sample of thickness *x* with attenuation coefficient µ.

In addition to their attenuation, as the X-rays pass through the sample, their phase can be altered based on variations in the refractive index within the sample. The refractive index, a complex term, has contributions based on both phase and absorption:
(2)

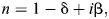

where n is the refractive index, δ accounts for the phase contribution and β for the absorption. β is related to µ in Eqn 1 via Eqn 3:
(3)

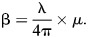

For soft tissue and X-rays in the range of 17–40 keV, δ is typically three orders of magnitude greater than the value for β. Therefore, phase-contrast X-ray imaging of tissue is far more effective than trying to image via absorption.

Moving these imaging techniques into three dimensions (3D) requires tomography. X-ray imaging tomography is performed by rotating the sample within the X-ray beam around at least one axis while a series of projection (still) images are acquired. Typically, the sample is rotated by at least 180° to ensure a full angular coverage. The series of 2D images is then phase retrieved and computationally reconstructed into a 3D dataset. The most commonly used method uses a filtered back projection algorithm (FBP). In simple back projection (BP), the attenuation profile at each rotation angle is ‘back-projected’ across image space. The attenuation value in the sinogram is divided by the number of image pixels along the direction of the projection from the X-ray source to detector, and the average attenuation value thus obtained is assigned to these pixels. In FBP, the projection images are filtered beforehand to reduce blurring in the reconstructed image (Schofield et al., 2020). However, algebraic or iterative methods are also used, although they can be more computationally expensive (Beister et al., 2012; Nilchian et al., 2013). Iterative reconstruction refers to an image reconstruction algorithm (see Glossary) used in CT that begins with an image assumption and compares it to real-time measured values while making constant adjustments until the two are in agreement.

Acquisition times vary across different setups. Synchrotrons drastically reduce single projection exposure times due to the high available photon flux. Standard exposure times per projection are in the range of 10–100 ms, with extreme examples using <1 ms (Mokso et al., 2015). In a parallel beam configuration, the scan times are in a range of <1 min to a few minutes. Generally, a high-resolution cone beam configuration lowers the photon flux and increases the scan times to a range of tens of minutes to hours. Although the scan times are often precisely described in literature, the processing times associated with the data are left out. Reconstruction is essential to view and analyse the data in 3D, and it is also a computationally complex and heavy process that is not easily performed by non-experts. Many synchrotron experiments result in terabytes of data that need to be reconstructed, a process often taking weeks after the experiment has finished. Therefore, we believe it is essential for the facilities to not only report on the estimated reconstruction times, but to deliver the reconstructed data to the user. This is something we have started to address here in Hamburg (Albers et al., 2024).

Although propagation-based phase-contrast X-ray imaging is the most commonly used method for biological samples, there are other ‘flavours’ of phase-contrast imaging that introduce additional optical elements to resolve the phase information. For further details and an exploration of other possible imaging techniques, please refer to Endrizzi (2018), which is an excellent review on the topic. In addition, hard X-ray ptychography, a coherent diffractive X-ray imaging technique, shows very promising results for high-resolution imaging of biological samples (Bosch et al., 2024 preprint; Cipiccia et al., 2024). Although ptychography techniques have achieved higher resolutions than holography, they are more limited in terms of field of view and sample thickness. However, for the scope of this Review, we will concentrate on propagation-based phase-contrast techniques.

### Preparation of biological samples for X-ray imaging

Tissue samples can be prepared for X-ray imaging in different ways. Currently, there is no strict sample preparation protocol designed exclusively for X-ray imaging. Rather, the community imaging at synchrotrons has both adopted and adapted the use of existing protocols practiced in clinical and research settings. This makes X-ray imaging a highly complementary technique; for example, it is readily compatible with the classical histological process of formalin fixation and paraffin embedding (FFPE) (Saccomano et al., 2018; Töpperwien et al., 2018b). Fixation, dehydration and paraffinisation enhance the innate tissue contrast while creating a stable solid medium which does not heat or move due to X-ray beam exposure. It is also possible to image samples that have been prepared for vEM – i.e. heavy metal-stained and resin-embedded samples (Albers et al., 2024; Bosch et al., 2022). Here, staining such samples with osmium tetroxide gives additional contrast to the tissue against the resin background. Both of these preparations are mounted in a solid format, and therefore the samples can be scanned multiple times without consequences. Imaging in liquid suspension and gels is performed less frequently, mainly due to potential sample movement from heating, bubble formation or gel deformation (Xian et al., 2024). In low-absorbing, unstained samples, this risk is minimised; nevertheless, careful considerations should be made when tuning the imaging parameters. Liquid samples benefit from lower beam intensity and higher X-ray energies, ensuring that X-ray interaction with matter is minimised; however, the quality of the images decreases substantially. Therefore, many choose to image serially dehydrated samples suspended in high concentrations of ethanol, which provides better stability than aqueous solutions. Although it is a strong fixative, ethanol dehydration of the tissue can be reversed by careful serial rehydration if needed. For large specimen imaging (see next section), agarose gel cubes infused with high-percentage ethanol are used (Brunet et al., 2023). This solution immobilises the specimen without the need to address the challenges associated with embedding very large samples. Nevertheless, tissue fixation does cause irreversible protein crosslinking and nucleic acid fragmentation, which are incompatible with many multi-omics applications. To address this issue, efforts are being made to enable X-ray imaging without the need for chemical fixation and using cryo-conditions instead (Maes et al., 2022, 2024). However, cryo-preserved samples are prone to ice-crystal formation within the tissue and movement due to thawing, both of which are detrimental to X-ray imaging. Therefore, depending on the nature and size of the tissue, cryo-SRµCT is a delicate and challenging technique. As a result, there are very few publications in this area. However, recent studies have reported successful imaging under cryo-conditions in unfixed snap-frozen cardiac biopsy samples, highlighting the potential of this approach for enabling multiscale and multi-method analysis (Li et al., 2023b).

## Biological applications for propagation-based synchrotron hard X-ray imaging

### Large specimens and efforts towards patient imaging

**Fig. 2.**
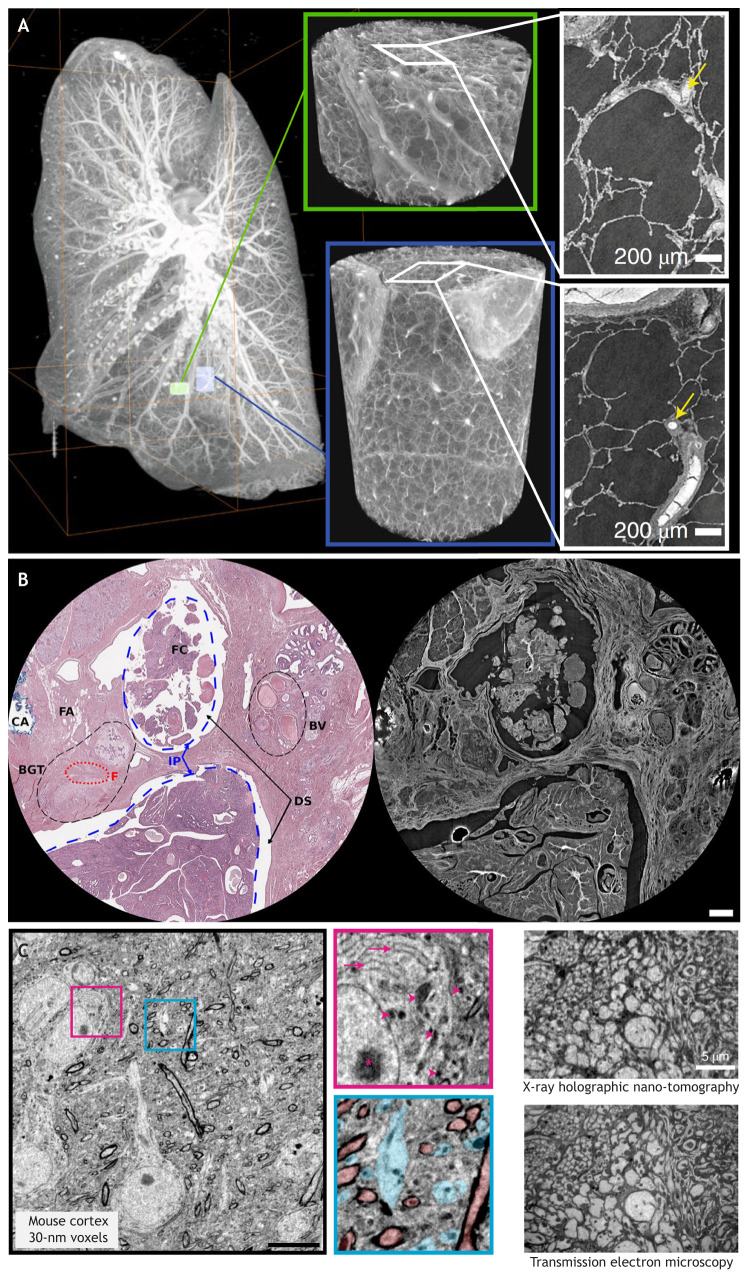
**Biological applications of SRµCT and SRnCT.** (A) Maximum intensity projection of a whole human lung imaged by HiP-CT with two randomly selected volumes imaged at a resolution of 2.45 μm per voxel highlighted in green and blue. 3D reconstructions of the two high-resolution VOI are shown with 2D slices in the insets. In the 3D high-resolution volumes, the fine mesh of pulmonary blood vessels and the complex network of pulmonary alveoli and their septa can be seen. Yellow arrows denote occluded capillaries in 2D slices. Adapted from Walsh et al. (2021) where it was published under a CC-BY 4.0 license. (B) Region of histological features of interest for intraductal papilloma as seen in conventional H&E staining (left) and SRµCT (right). Abbreviations: intraductal papilloma portion (IP), ductal space (DS), blood vessels (BV), fibroadipose tissue (FA), elastic collagen and fibres (F), breast glandular tissue (BGT), cauterisation artifacts (CA), fibrovascular cores (FC). The scalebar is 0.5 mm. Adapted from Donato et al., 2024a where it was published under a CC-BY 4.0 license. (C) Virtual slice through a higher-resolution SRnCT volume of mouse primary somatosensory cortex (30-nm voxels). Insets: detailed views showing ultrastructural features, including mitochondria (magenta arrowheads), endoplasmic reticulum (magenta arrows), nucleolus (magenta asterisk), large dendrites (cyan) and myelinated axons (red). Scale bar: 10 μm (areas marked in blue and pink are 10 μm in width). Reproduced from Kuan et al. (2020) with permission from Springer Nature.

Perhaps one of the most notable projects to utilise synchrotron X-ray tomography in recent years is hierarchical phase-contrast tomography (HiP-CT) (Ackermann et al., 2022; Walsh et al., 2021; Xian et al., 2022). By taking advantage of a highly coherent X-ray source, HiP-CT has been performed on fixed whole human organs across a range of resolutions, starting at the whole-organ level with a voxel size of 25 µm and progressively zooming in with a final voxel size of 1.3 µm. This process was made possible through a careful selection and calibration of the beamline hardware components, sample preparation and mounting. HiP-CT is performed using a polychromatic beam in edge detection mode using two different detectors for the voxel ranges. The authors have further demonstrated the computational potential of the data by analysing the alveolar thickness and porosity of a COVID-19-exposed lung (Walsh et al., 2021) (Fig. 2A). However, one of the bigger achievements of the project is the detailed multiresolution data made available to the public. The ability to view a whole human organ in high detail and in 3D with or without pathology has great value not only in disease research but in medical and general education. The Human Organ Atlas (https://human-organ-atlas.esrf.eu/) initiative is aimed at creating a database of such organs and make them easily accessible.

Although HiP-CT can resolve large samples, such as whole human organs, at an unprecedented resolution, due to the huge amount of required X-ray dose (see Glossary), HiP-CT is limited to *ex vivo* or post-mortem analysis of samples. Another approach for large specimen imaging has been demonstrated using a fresh pig lung encased in a plastic phantom that mimics a human chest. The phantom consists of a double-walled plastic chamber that houses a fresh lung, which is inflated via negative pressure applied to the phantom chamber, ensuring a physiologically compatible inflation (Wagner et al., 2018). More recently, an updated phantom that incorporates an artificial rib cage and a mobile diaphragm has been developed to better mimic the human situation (Dullin et al., 2024). Using this setup and SRµCT on acute respiratory distress syndrome (ARDS) disease model lungs, the authors show that even heterogeneous distension of individual alveolar air spaces can be resolved (Albers et al., 2023). This setup opens up the possibility of studying rare and complex infectious lung diseases in a near-native state, provided that appropriate safety measures are in place. Additionally, this setup has been tested as a proof of concept for patient imaging. A major concern in patient X-ray imaging is the deposition of dose (see Glossary). Phase-contrast imaging is a lot more dose efficient than regular attenuation imaging, as far fewer X-rays are absorbed by the sample. A seven-fold improvement in resolution from-state-of-the-art clinical CT to SRµCT was demonstrated at the same dose levels. Additionally, phase contrast allowed better differentiation of relevant lung features, such as solid and sub-solid nodules, while remaining at clinically acceptable radiation dose levels (Albers et al., 2023). However, it should be noted that due to the limited vertical height of a synchrotron beam, only a small cross-section of the lung can be imaged per step. Furthermore, although the phantom effectively mimics lung conditions, including breathing motion, the absorption of X-rays by muscle and fat is not yet accounted for. Before a patient trial can be considered, further testing is required. With its superior resolution and contrast, phase-contrast imaging has the potential to improve diagnostics and reveal disease features not resolvable by current clinical imaging methods.

### 3D virtual histology

One of the challenges in the development of 3D imaging and its application to a new user community is to encourage thinking in 3D when many current applications are limited to 2D. Histological assessment is a gold standard in both preclinical and clinical research due to high specificity, speed and low cost. Nevertheless, it remains inherently 2D. Understanding the 3D context becomes crucial in unravelling disease development and the corresponding alterations. Structures such as vessels, lymph nodes, tumour architecture and infiltration fronts cannot be fully appreciated using 2D representation. Virtual histology is an emerging imaging technique that introduces high-resolution true isotropic 3D imaging to overcome the 2D representation of 3D structures (Fig. 2).

A well-explored branch of virtual histology is imaging of readily available clinical biopsy samples processed by FFPE. FFPE is a standard protocol in both clinical and preclinical practice performed for tissue preservation, subsequent sectioning and histological staining, and further archiving of the sample in a stable environment. Phase-contrast X-ray imaging allows data to be collected on both stained and unstained tissue and in a non-destructive manner and is thus, compatible with subsequent histology and immuno-histochemistry workflows. Owing to the geometry and size of the standard FFPE block, imaging in-block and/or of the entire block poses many challenges. Local tomography in the regions of interest (ROIs) inside the whole FFPE block can be prone to artefacts created by the highly absorbing plastic cassette that is used as the base of the FFPE block.

Imaging of the entire FFPE block can be made possible by acquiring slightly overlapping tomograms that can be stitched together after the reconstruction. Although stitching can help cover a much larger field of view (and thus retaining the effective voxel size), it tends to generate very large datasets that are difficult to work with. Provided that the horizontal range of the paraffin block is covered by a single scan, using vertical stitching alone, the whole FFPE block can be obtained in five to six vertical steps at a voxel size of 2 µm (Dullin et al., 2021; Lee et al., 2024). This has been reported in rat lung fibrosis and human colon carcinoma samples (D'Amico et al., 2024; Svetlove et al., 2023). An alternative to tomogram stitching is stitching in projection space before tomographic reconstruction. This approach has been recently demonstrated on a whole fixed and dehydrated mouse brain, which was imaged with an effective voxel size of 0.650 µm using only four off-centre acquisitions. The tomogram of the whole brain was successfully merged with the existing Allen Mouse Brain atlas (https://mouse.brain-map.org/). This volume is currently the most complete and comprehensive morphological representation of a whole mouse brain, a feat that cannot be achieved by any other imaging technique (Humbel et al., 2024 preprint).

It is also possible to perform local tomography of an FFPE block by extracting the ROI from the block mechanically before imaging. This is routinely performed by either manual cropping and extraction of a paraffin volume with a scalpel, or by using clinical tools, such as biopsy punches (Eckermann et al., 2021b; Salg et al., 2024). The latter approach comes with the advantage of having a variety of standardised diameters (1–8 mm) and cylindrical geometry, both of which are exceptional for tomography. Imaging in cropped paraffin volumes has been extensively demonstrated in a variety of clinical and preclinical samples, many of which have shown a morphological correspondence between virtual cutting planes in the SRµCT dataset with haematoxylin and eosin (H&E) histological sections. This approach allows a structure in a 3D SRµCT dataset to be traced while having an exact functional reference from the histological staining. Using this method, one study followed the spatial distribution of micropapillary intracystic and invasive lobular carcinomas in breast samples (Donato et al., 2024a) (Fig. 2B). Similarly, it has been shown that, using a single histological slice as a guide, features such as vessels, muscle, fat, ducts and tumours can be segmented in 3D from tomography datasets (Albers et al., 2021; Frohn et al., 2020; Pinkert-Leetsch et al., 2023).

SRµCT can be used as a stand-alone tool in cases where structures are easily identifiable from morphological features (e.g. vascular, bronchial, alveolar and brain infrastructures) or where a less morphologically diverse tissue is investigated (e.g. myosin fibre arrangement in heart tissue). The following examples demonstrate the capabilities of virtual histology and its importance in studying disease in 3D. In the case of cervical and thyroidal samples, one study uncovered a clearly delineated basal membrane in the SRµCT volumes, a feature that was not prominent in the histological sections (Donato et al., 2024b). The irregularity of the basal membrane here allowed the researchers to catch a transition between a pre-cancerous state and an early invasion. Alteration in vessel architecture in cardiac tissue derived from an individual with COVID-19 was recently studied with SRnCT. Drastic changes in the diameters of the vascular tree and pillar formation within were observed, indicating an intussusceptive angiogenesis process (Reichardt et al., 2021). Reichmann et al. were the first to visualise in 3D the channel of Lambert, an important collateral ventilation feature (Reichmann et al., 2023a). Using SRµCT imaging, another study has found that cerebellum samples of individuals with multiple sclerosis show an increased density of neurons and increased heterogeneity of the neuronal nuclei (Frost et al., 2023). A similar trend was reported on in samples from an individual with Alzheimer's disease where SRµCT uncovered increased heterogeneity of the granule cell nuclei (Eckermann et al., 2021a). Yet another study has shown that corpora amylacea generates excellent contrast in SRµCT and its distribution can be well studied with respect to vessels and cerebrospinal fluid (Lee et al., 2023). Finally, the power of virtual histology was demonstrated when the disorganisation of the local myocardium in cardiac samples from individuals with advanced heart failure was investigated and quantified in 3D (Li et al., 2023c).

Despite its clear success in pre-clinical and research settings, virtual histology is yet to become available in the clinical routine. For clinical histology, key factors include cost, speed and variety of available stains. At present, to come close to achieving the speed of histological processing needed in a clinical setting, a synchrotron is required. Recently, a unique high-throughput setup was developed, with acquisition and reconstruction of a single 1.3 mm field-of-view tomogram achieved in under 4 min using highly efficient reconstruction algorithms (Albers et al., 2024). There are also developments taking place to optimise laboratory-based X-ray systems dedicated to 3D histological analysis that could be located in a clinical setting. SRµCT-generated contrast cannot be compared to the range of structure-specific histological stainings. Efforts are being made to develop structure-specific dyes that can be used in the X-ray imaging setting using a variety of approaches including metal-conjugated antibodies. So far, stains for nuclei (Metscher, 2021; Müller et al., 2018), cytoplasm (Busse et al., 2021) and a range of non-specific enhancing stains exist (Metscher, 2009; Reichmann et al., 2023b; Saccomano et al., 2018; Xia et al., 2020). Nevertheless, 3D virtual histology remains an indispensable method in multidisciplinary imaging and is foreseen to become a valuable tool in the clinical practice.

### Correlative X-ray imaging – combining synchrotron X-ray imaging with other imaging modalities

X-ray imaging in the home laboratory has often been used as a method to identify an ROI for a higher resolution but lower field of view data collection, such as vEM (see, for example, Cheng et al., 2020). However, X-ray imaging and vEM work exceedingly well together, particularly in lab-based settings, given that the addition of metal staining to the sample, which is essential for EM data collection, results in the ability to collect interpretable data with a laboratory X-ray source. There is increasing interest in taking the data collection on these projects to the synchrotron for a variety of reasons; synchrotron-based imaging is typically very quick, enabling many samples to be screened for, e.g. for capturing rare events. Additionally, the very precise targeting of EM data collection based on the higher resolutions achievable at the synchrotron cuts down on EM microscope acquisition time, which increases the throughput within the EM laboratory as well. An early example linking synchrotron-based X-ray imaging with EM was carried out at the Italian synchrotron Elettra (Parlanti et al., 2017). That study demonstrated that a high-resolution 3D volume acquired with SRµCT can be used to identify focal disease features that can be precisely targeted for subsequent investigation with an even higher resolution vEM. However, this work went relatively unnoticed for a number of years – possibly associated with the low-profile nature of vEM techniques until they hit the headlines when they were identified by Nature as one of the techniques to watch in 2023 (Collinson et al., 2023; Eisenstein, 2023), the so-called ‘silent revolution’.

Using X-ray volume datasets as a targeting ground for other downstream techniques, such as vEM, is an excellent approach. A correlative workflow linking 3D X-ray imaging data with classical histological data and atomic force microscopy (AFM) was recently developed and utilised to study lung fibrosis development (D'Amico et al., 2024). The propagation-based X-ray imaging identified ROIs in the mouse lung, which was used for precision slice cutting to perform histology and Young's modulus measurements with AFM. Based on the X-ray data, alveolar porosity calculations quantitatively demonstrated increased tissue density and reduced alveolar space, while histology and AFM showed differences in collagen distributions between the two mouse models. Together, this comprehensive correlative approach allowed for differentiation of the modes of induction of pulmonary fibrosis in a mouse model. Owing to the versatility of the synchrotron X-ray imaging and a multitude of possibilities for comprehensive correlative studies, it can be confidently predicted that more work will be published, demonstrating the development and growth of this area, as well as greater recognition of the power of this technique.

### Pushing the resolution limits – ultra high-resolution X-ray imaging

An incredibly powerful advantage that X-rays have over other imaging modalities is their penetrative properties, along with the ability to image down to resolutions below 100 nm. Although most synchrotron setups for biological X-ray tomography utilise the parallel X-ray beam provided by the light source (see Glossary), this limits the obtainable resolution. Parallel beam geometry has many advantages as it can make use of the full coherence of the X-ray beam, allowing phase-contrast imaging, while at the same time simplifying the 3D reconstruction problem. The downside is that there is no geometric magnification. Lab-based X-ray imaging systems utilise small spot sizes of the X-ray source, producing a cone beam, resulting in a magnified image of the sample on the detector. To achieve very high resolution, there are many approaches combining the powerful X-ray beam a synchrotron offers with a cone beam geometry. Techniques reaching resolution levels in a (low) nanometer range are termed ‘nanotomography’ – where nano relates to the field of view and high resolution rather than a very small amount of tomography (Fig. 1F). Unsurprisingly, imaging at this scale is a challenge. Different X-ray beam focusing optics can be used including elliptically curved Kirkpatrick–Baez (KB) focusing mirrors (Silva et al., 2017), Fresnel zone plates (Flenner et al., 2022) or X-ray waveguide optics (Salditt et al., 2015). The drawbacks of these approaches are the small field-of-view and scanning times that are much longer than in parallel beam configurations. The power of these high-resolution setups has been demonstrated, among others, by one study that characterised neuronal depositions like Lewy bodies, Hirano bodies and β-amyloid plaques in human brain tissues (Reichmann et al., 2024 preprint). Further examples include the localisation of macrophages in mouse lungs (Krenkel et al., 2015), human cartilage in osteoarthritis formation (Horng et al., 2021) and Langerhans islets in human pancreatic tissues (Frohn et al., 2020).

### Resolving the connectome of the brain

One major driver to push the resolution limits in X-ray imaging is the desire to solve the connectome of the brain. Connectomics describes the field of science that tries to unravel the different connections between the neurons in the brain, effectively trying to draw a circuit diagram that might provide a greater level of understanding of how the brain functions. Currently, vEM is the only imaging method that can reliably produce 3D data sets with a synaptic resolution of <10 nm. But vEM in itself is a slow and destructive method that requires sectioning of the specimen either prior to or during the data acquisition. For example, a recently published study produced a 1.4 petabyte dataset of a human brain fragment imaged with a multi beam scanning electron microscope. The acquisition time for one sample with a volume of ∼1 mm^3^ and a voxel size of 4 nm was 326 days (Shapson-Coe et al., 2024). In contrast, another study demonstrated the capabilities of ultra-high resolution SRnCT on neuronal tissues of both *Drosophila* and mouse (Kuan et al., 2020) (Fig. 2C). Here, the authors successfully reconstructed the axonal connectivity of both cortical pyramidal cells, as well as traced back individual motor axons from the muscles in a *Drosophila* leg to the central nervous system of the fly.

There is a hope that new advances in the field of SRnCT and with the emergence of fourth-generation synchrotron light sources, that X-rays can be an important player in deciphering the connectome of the brain (see report by Jefferis et al., 2023 at https://wellcome.org/reports/scaling-connectomics).

## Bottlenecks and challenges – data handling

Like other high-resolution imaging techniques, phase-contrast X-ray imaging utilises state-of-the-art hardware to record as much detail as possible. This typically results in terabytes of reconstructed data from a single day of synchrotron measurements. Exploration of such data is made difficult by the common lack of powerful enough machines in daily research use. Multi-terabyte volumes require at least the same amount of rapid access memory (RAM) and a powerful graphics card in a standard monolithic file format for effective visualisation and manipulation of the data. This issue has been tackled by the development of next-generation file formats (NGFFs), which use a pyramidal structure to display only a part of image without loading the entire dataset. Electron and light microscopy communities recently standardised and built upon the NGFF Zarr structure to develop the Open Microscopy Environment (OME)-Zarr. OME-Zarr has a transparent organisation of image chunks that store multiple resolution levels (Moore et al., 2023).

One other challenge is to make sure that all data adopt the FAIR principle (‘findable, accessible, interoperable and reusable’). Besides displaying and sharing large datasets, making them publicly available is the next challenging step. Furthermore, with the increased interest in multimodal imaging, often data on a single sample is available from many methods and requires co-registration. Currently, multiple platforms that enable display of multi-modal imaging data are under development by major scientific institutions. The Multimodal Big Image Data Exploration (MoBIE) plug-in for the well-established program Fiji is a perfect example of a development resulting from the need to visualise huge amounts of data without needing to download terabytes (Pape et al., 2023). Other similar applications include Webknossos (Boergens et al., 2017), Neuroglancer (https://github.com/google/neuroglancer) and Napari (Chiu et al., 2022; Perkel, 2021). Many of these platforms integrate the ability to stream data from a cloud service. This, in combination with the standardised OME-Zarr formats, brings the field closer to achieving the FAIR data principle with experts in different disciplines able to interrogate the data for their own purpose from the comfort of their own space. However, collective community action is still required to create a standardised system for data viewing and sharing. To date, commonly used 3D rendering software such as Dragonfly, Amira and VG Studio Max cannot import, export or stream NGFF formats.

A widely spoken of aspect of X-ray imaging is its advantage in 3D feature depiction. Rendering of individual features propagated in 3D space (e.g. vessels) requires instance segmentation. Although manual segmentation of X-ray datasets can be easily performed, it is labour intensive and requires an expert to determine the correct boundaries. In order to make the most out of the data we can collect, we need to invest heavily in the development of automated segmentation routines. Fortunately, this field is active. Less ideal is the fact that X-ray data, particularly of unstained tissue samples, is relatively weak in contrast. Many artificial intelligence automated segmentation routines are trained either on 2D microscopy or 3D EM data (Archit et al., 2023 preprint; Stringer et al., 2021). vEM images carry similar features to X-ray imaging data, therefore it is worth investing in adapting existing approaches to X-ray data. Some progress has been made in this regard – a 3D U-net convolutional neural network developed for EM has been adapted to X-ray data (Kuan et al., 2020) and developed further via a data challenge (https://xpress.grand-challenge.org/). Easy segmentation tasks, such as nucleus segmentation, have been performed using machine learning in X-ray data (Frost et al., 2023).

Overall, it is worth taking the time to consider whether there is merit in trying to develop a coherent strategy for data handling analysis and sharing. It would be a huge, but worthwhile, challenge.

## Conclusions and future prospects

The use of X-ray imaging as a targeting tool in, for example, a vEM workflow is now established primarily through published works using laboratory-based X-ray data. By moving the X-ray data collection from the laboratory to the synchrotron, both the quality of the data and the speed with which it can be obtained are significantly improved. Both of these allow a large number of samples to be imaged, which has two benefits: firstly, it enables statistically sound population studies, allowing the endemic variability in biology to be better demonstrated. Secondly, downstream complex and time-consuming techniques, such as vEM, can be performed on carefully selected ROIs, making an otherwise impossible task more manageable.

The ability to image tissues that are fixed but suspended in liquid is significant; it presents the opportunity to carry out genomic analyses on tissues post-data collection. It has been established that the X-ray data collection does not destroy the genetic information contained within the cell (Li et al., 2023a). The future steps include combining X-ray imaging with, for example, spatial transcriptomics and/or immunofluorescence and other similar techniques to gain a multiplexing detailed view of the tissue as a whole. The acquisition of this information brings the potential of targeted personalised medicine a step closer.

Nevertheless, segmentation of features of interest and their analysis remains a hurdle for many comprehensive imaging studies. There is significant investment in harnessing artificial intelligence techniques, taking advantage of developments in other imaging modalities such as light and electron microscopy (Cunha et al., 2024). There are of course challenges, such as in instances where samples are imaged hydrated in liquid meaning the contrast of the X-ray data is particularly low. Although the eye–brain interface is adept at identifying boundaries between features, this is computationally challenging. Additionally, the existing methods being built on primarily function in a 2D area or a 2D+ volume, whereas the X-ray imaging data extends isotropically in 3D. However, progress is being made, and the more X-ray imaging is used on complex biological samples, the more data can be made available to improve the matter.

Overall, the combination of the significant phase component of the synchrotron beam, especially in the latest fourth generation sources (Chapman, 2023), with visionary biologists willing to explore new imaging methods, means that biological X-ray imaging of soft biological tissue is on the cusp of an exponential rise in popularity helped particularly by the speed of high-quality data acquisition.
